# Nitrogen in the defense system of *Annona emarginata* (Schltdl.) H. Rainer

**DOI:** 10.1371/journal.pone.0217930

**Published:** 2019-06-06

**Authors:** Felipe Girotto Campos, Maria Aparecida Ribeiro Vieira, Amanda Cristina Esteves Amaro, Iván delaCruz-Chacón, Marcia Ortiz Mayo Marques, Gisela Ferreira, Carmen Sílvia Fernandes Boaro

**Affiliations:** 1 Instituto de Biociências, UNESP: Universidade Estadual Paulista, Campus Botucatu, Departamento de Botânica, Botucatu, São Paulo, Brazil; 2 Faculdade de Ciências Agronômicas, UNESP: Universidade Estadual Paulista, Campus Botucatu, Departamento de Horticultura, Botucatu, São Paulo, Brazil; 3 Laboratorio de Fisiología y Química Vegetal, Instituto de Ciencias Biológicas, Universidad de Ciencias y Artes de Chiapas (UNICACH), Tuxtla Gutiérrez, Chiapas, Mexico; 4 Centro de Pesquisa de Recursos Genéticos Vegetais, Instituto Agronômico (IAC), Campinas, São Paulo, Brazil; Ankara University, TURKEY

## Abstract

The concentration of nitrogen can generate different strategies in plants in response to stress. In this study, we investigated how nitrogen concentration interferes with the defense system of *Annona emarginata*. Low concentrations of nitrogen increased the allocation of photosynthetic resources to carbon metabolism, resulting in an increase in the synthesis of volatile substances involved in signaling and defense that contributed to antioxidant enzymes in overcoming stress. The availability of nitrogen at 5.62 mM concentration might have helped to induce increased resistance in the plants because at this concentration, signaling substances and defense substances (monoterpenes and sesquiterpenes) were observed. Plants cultivated with the highest nitrate concentration displaced energy for the reduction of this ion, likely forming nitric oxide, a signaling molecule. This condition, together with the decrease in carbon skeletons, may have contributed to the lower synthesis of volatile substances of the specialized metabolism that are also involved with signaling. Varying the nitrogen in *Annona emarginata* cultivation revealed that depending on the concentration, volatile substances show higher or lower synthesis and participation in the system of signaling and defense in the plant. These results may suggest that volatile substances participate in resistance to pests and diseases, which is a necessary condition for *Annona emarginata* to be preferentially used as rootstock for *Annona* x *atemoya*.

## Introduction

Plants require high amounts of nitrogen (N) because this element is involved in the biosynthesis of substances of the primary and specialized metabolisms [1]. In these metabolisms, substances formed during photosynthesis and nitrogen assimilation are converted into proteins, nucleic acids, lipids, chlorophyll, phenylpropanoids, flavonoids, terpenes, and alkaloids, which are important for adaptation to biotic and abiotic stresses [2].

Plants grown under low N levels do not express their complete genetic potential, which can interfere with their physiology [3]. N deficiency may cause altered allocation of assimilated carbon (C), leading to changes in the amounts of leaf starch, sucrose, and monosaccharides [4,5] and reductions in the amount and carboxylation activity of Rubisco. The ribulose-1,5-bisphosphate carboxylase/oxygenase (Rubisco) enzyme requires 30% N for its synthesis [6–8]. Thus, N deficiency can affect the biochemical phase of photosynthesis, altering the availability of carbon skeletons [9] used in the synthesis of carbohydrates and amino acids [4]. However, high N levels can also affect carbon metabolism, particularly when N is in the form of nitrate, which requires reducing agents for its assimilation, thereby competing with the assimilation of C [2,10].

The concentration and source of N are critical for a plant to develop effective defenses against pathogens. Nitrate may improve plant defenses through the signal molecule nitric oxide (NO) [11]. The enzymes peroxidase (POX), superoxide dismutase (SOD) and catalase (CAT) may indicate increased accumulation of reactive oxygen species (ROS), which signals growth processes, thereby accelerating the growth of plants [12]. *Catharanthus roseus* cultivated with nitrate showed increased activity of POX and SOD, and when the source was ammonium, CAT activity increased [13].

Increased activity of superoxide dismutase, peroxidase, and catalase control reactive oxygen species, which interfere with the maintenance of membrane integrity through lipid peroxidation. However, the presence of ROS acts on physiological processes such as growth and signaling for the production of defense molecules [14–16].

Reactive oxygen species not controlled by the enzymatic antioxidant system can trigger signals that intensify the production of molecules of the specialized metabolism, helping to neutralize and prevent structural damage at the membrane level [16–18].

Volatile substances are a mix of terpenes, phenolic molecules, and fatty acid derivatives, among others, which may vary in number and quantity when plants are subjected to climatic and nutritional changes and according to the phenological phase of the species at a given time [19]. Mono- and sesquiterpenes may be involved in plant defense pathways against biotic and abiotic stress [20,21] and aid the antioxidant enzymes in the neutralization of reactive species [22]. Fatty acid derivatives are also involved in signaling events of stress [23] and may be altered by variations in the concentration of nitrogen. Low nitrogen availability can signal stress, triggering the biosynthesis of substances involved in overcoming stress, such as fatty acid derivatives and jasmonic acid [1,23–25].

The incorporation of nitrogen and the biosynthesis of terpenes, including sesquiterpenes and monoterpenes, share the same reaction sites and are potential competitors for carbon skeletons and reducing agents [26]. Thus, variations in the concentration of nitrogen can result in changes in the availability of carbon skeletons and reducing agents that are used in the biosynthesis of hydrocarbons [4,27].

The biosynthesis of monoterpenes occurs via the 2-C-methylerythritol 4-phosphate pathway in plastids, particularly in the chloroplasts. Sesquiterpene biosynthesis occurs via the mevalonic acid pathway in the cytoplasm [28]. The biosynthesis of fatty acid derivatives also occurs in the plastids through the lipoxygenase (LOX) and hydroperoxide lyase (HPL) pathways [1].

The process of nitrate reduction to ammonia and incorporation requires much energy [4]. In this reduction, the conversion of nitrate to nitrite occurs in the cytoplasm by the action of the enzyme nitrate reductase. Subsequently, the nitrite is reduced to ammonia in the chloroplast by the enzyme nitrite reductase. NH_4_ is incorporated by the enzymes glutamine synthetase and glutamate synthase into carbonic chains through reactions that require reducing agents generated during the acyclic photochemical phase of photosynthesis [7,27].

Thus, the availability of nitrogen can influence the dynamics of specialized metabolite expression and the release of volatile substances, an important condition in plant-animal relations [29]. Plants of *Zea mays* var. Delprim, which are preyed on by the beet armyworm, *Spodoptera exigua*, showed an increase in the release of volatiles as the nitrogen supply decreased [30]. The addition of nitrogen to cotton plants decreased the concentration of leaf volatiles, and the larvae of *S*. *exigua* and *Cotesia marginiventris* (Cresson) were not repelled [29].

The biosynthesis of these substances depends on the availability of carbon (C), nitrogen (N) and energy supplied by the primary metabolism. As a consequence, the availability of these building blocks greatly affects the concentration of a specialized metabolite, demonstrating a high degree of connectivity between the primary and specialized metabolisms [1].

Nitrate may interfere with primary metabolism, and whether availability is high or low will influence the specialized metabolism and defense system. Thus, adequate concentrations can cause the production of signaling molecules that allow plants to acclimatize and overcome stress. When availability is low, the targeting of resources to the production of specialized metabolites can also assist in plant defense.

*Annona emarginata* has physical and chemical characteristics that make it resistant to various pests and diseases and, due to its compatibility with the hybrid *Annona* x *atemoya*, which produces fruit with high commercial value, is used as its rootstock [31]. Volatile substances should be highlighted among the chemical characteristics that contribute to the strength of *A*. *emarginata* rootstock. The concentration of nitrogen can influence primary metabolism, such as hydrocarbon biosynthesis, which generates the raw material for the synthesis of volatile substances. Nitrogen concentration variation can help to show how this element contributes resistance to pests and diseases of *A*. *emarginata*.

In this study, we investigated how nitrogen concentration influences interferes with the defense system of *Annona emarginata*.

## Materials and methods

### Plant species and N concentrations

Seedlings of *Annona emarginata* (Schltdl.) H. Rainer variety ‘terra-fria’ were brought from São Bento do Sapucaí, São Paulo, Brazil, which is located at 45°44′11″ W, 22°41′18″ S and 874 m above sea level. The seedlings were grown in a paddy-fan greenhouse at 26 ± 4°C located at 48°24′35′′ W, 22°49′10′′ S and 800 m above sea level at the Instituto de Biociências, São Paulo State University, Campus Botucatu, São Paulo. The experiment was a factorial design with four N levels and five harvests.

The plants were grown in 6-L pots containing Hoagland & Arnon’s nutrient solution n^o^. 1, using nitrate as the source of nitrogen [32] at 7.5 mM N, modified to provide N levels of 5.62, 3.75, and 1.87 mM (S1 Table). Plants were maintained until harvest at 150, 164, 172, 192, and 206 days after beginning treatment (DBT).

The temperature and relative humidity recorded in the greenhouse during the gas exchange evaluations are shown in S2 Table.

### Gas exchange

Gas exchange was measured from 9:00 a.m. to 11:00 a.m. using a LI-6400 portable photosynthesis system with an infrared radiation analyzer for CO_2_ and water vapor [Infrared Gas Analyzer (IRGA); Li-Cor Inc., Nebraska, USA]. The measurements, which were performed on the 2^nd^ or 3^rd^ leaves with completely expanded blades, were used to obtain estimates based on the difference between the air CO_2_ and water vapor levels for the reference and those of the sample. The levels of water vapor and CO_2_ released and assimilated by the leaf stomata were then determined.

The CO_2_ assimilation rate (*A*_*net*_, μmol CO_2_ m^−2^ s^−1^), transpiration rate (*E*, mmol water vapor m^−2^ s^−1^), and stomatal conductance (*g*_*s*_, mol m^−2^ s^−1^) were determined. Water use efficiency [WUE, μmol CO_2_ (mmol H_2_O^−1^)] was calculated using the ratio between CO_2_ assimilation and transpiration rates (*A*_net_/*E*). The apparent carboxylation efficiency was calculated according to the ratio between the CO_2_ assimilation rate and the intercellular CO_2_ concentration of the leaf (*A*_*net*_/*C*_*i*_, mol m^−2^ s^−1^ Pa^−1^) [33].

The photosynthetic potential was evaluated using CO_2_ response curves (*A*_*net*_/*C*_*i*_ curves) [34] at 192 DBT under a saturating irradiance of 1,200 μmol m^−2^ s^−1^ photons, in accordance with a previous experiment on this species. The curves were fitted according to the Sharkey model [35] after calculating the maximum carboxylation rate of ribulose-1,5-bisphosphate carboxylase/oxygenase (*V*_*cmax*_, μmol CO_2_ m^−2^ s^−1^), the photosynthetic electron transport rate (*J*_*max*_, mmol electrons m^−2^ s^−1^), and the respiratory rate (Rd*, μmol CO_2_ m^−2^ s^−1^).

### Net assimilation and relative growth rate

The plants were separated into leaf blades, stems plus petioles, and roots. After determining the leaf area, leaf weight, and total dry weight, the physiological indices net assimilation rate (NAR) and relative growth rate (RGR) [36] were determined following Portes and Castro Jr. [37].

### Nitrate reductase activity

For the measurement of nitrate reductase activity, leaves were kept on ice until the analysis, as described by Jaworski [38].

### Leaf carbohydrate and total amino acid concentrations

The extraction of total soluble sugars was performed as described by Garcia *et al*. [39] with minor modifications, and starch extraction was conducted according to Clegg [40].

The procedure for determining the concentration of total soluble sugars was conducted according to Morris [41], that for starch as described by Yemm and Willis [42], that for reducing sugars as determined by Miller [43], that for sucrose as established by Passos [44] and that for free amino acids as described by Yemm and Cocking [45].

### The activity of antioxidant enzymes and lipid peroxidation

The extraction of antioxidant enzymes was performed as described by Kar and Mishra [46]. The activities of the enzymes superoxide dismutase, EC 1.15.1.1, and catalase, EC 1.11.1.6, were determined by the method of Peixoto et al. [47]; the activity of peroxidase, EC 1.11.1.7, was established according to Teisseire and Guy [48]; and the total soluble proteins were quantified as described by Bradford [49].

Lipid peroxidation was determined according to Heath and Packer [50], according to Devi and Rama Prasad [51].

### Analysis and identification of the volatile substances

Dried leaf blades were used for the extraction of volatiles in plants subjected to different concentrations of nitrogen to determine the volatile substance profile.

For this purpose, the leaves were dried in an air-flow oven at 40°C. The volatiles were captured by headspace solid-phase microextraction mode with an SPME Fiber Assembly [75 μm of carboxen/polydimethylsiloxane (CAR/PDMS)] for use with a manual holder (Supelco). Leaves (250 mg of dry mass) were placed in a glass vial (10 mL) with distilled water (5 mL), and the vial was sealed. The mixture was heated in a water bath at 90°C for 1 h. After this period, the fiber was exposed to the headspace for 15 min. The volatiles were immediately desorbed at 220°C, separated and detected using gas chromatograph coupled to a mass spectrometer (GC-MS-Shimadzu, QP-5000) GC-MS.

The chemical composition of the volatile substances extracted from the leaves was determined by GC-MS (Shimadzu, QP-5000) with electron impact (70 eV); detector at 230°C; a fused silica capillary column, DB-5 (30 m × 0.25 mm × 0.25 μm); helium as the carrier gas (flow 1.0 mL min^−1^); a temperature program of 60°C (2 min) and then 60–240°C at 3°C min^-1^; a split ratio of 1/20; and a flow rate of 1 mL min^−1^. The identification of substances was performed by comparison of their mass spectra with the GC-MS system database (Nist. 62 Libr.), literature [52] and retention indices (RI). To obtain the RI of the substances, a mixture of *n*-alkanes (C_9_-C_24_; Sigma Aldrich 99%) was employed and analyzed under the same operating conditions as those of the samples, and the Van den Dool & Kratz equation was used [53].

All procedures were conducted from 9:00 a.m. to 11:00 a.m. with completely expanded leaves.

### Statistical analyses

The data were subjected to analysis of variance (ANOVA), and means were compared using Tukey’s test at the 5% probability level; regression analysis was also performed. Using the SAS statistical software package 9.2 (SAS Institute Inc., Cary, NC), Levene’s test was used to test the homogeneity of variances in the treatments [54].

The effects of nitrogen on available volatiles were analyzed in relation to the number of molecules in the profile (profile density) in general or based on the group to which the substances belonged (monoterpenes, sesquiterpenes, and fatty acid derivatives) and in relation to the relative abundances of these substances. The profile of the volatiles was also expressed as the phytochemistry diversity index using the Shannon index (H).

The diversity index of the total volatiles (signaling and defense substances (mono- and sesquiterpenes)) was calculated using the following formula: H = −Σ (pi ln pi), where pi = the proportion of substance i in relation to the total volatile substances (relative abundance of the substance i) calculated by ni/N, where ni = the relative percentage of substance i and N = the sum of the percentage of total substances.

The diversity indices (H index) and the number and relative percentages of substances were subjected to ANOVA, and the means were compared using Tukey’s test at the probability level of 5%. Using SigmaPlot 12.0 (Systat Software, Inc. SigmaPlot for Windows), Levene’s test was applied to test the homogeneity of variances across treatments.

Principal component analysis (PCA), Pearson’s correlation coefficient and hierarchical cluster analysis (HCA), and similarity and Pearson’s coefficient (UPGMA) were performed with the relative percentages for substance identification at the different nitrogen concentrations and evaluation time points, and a different hierarchical cluster analysis (Ward’s algorithm, index of dissimilarity of Euclidian distance) was performed using the values of the chemical diversity at the four concentrations of nitrogen and evaluation time points by the software XLSTAT (2017).

## Results

### Gas exchange

**Fig 1:**
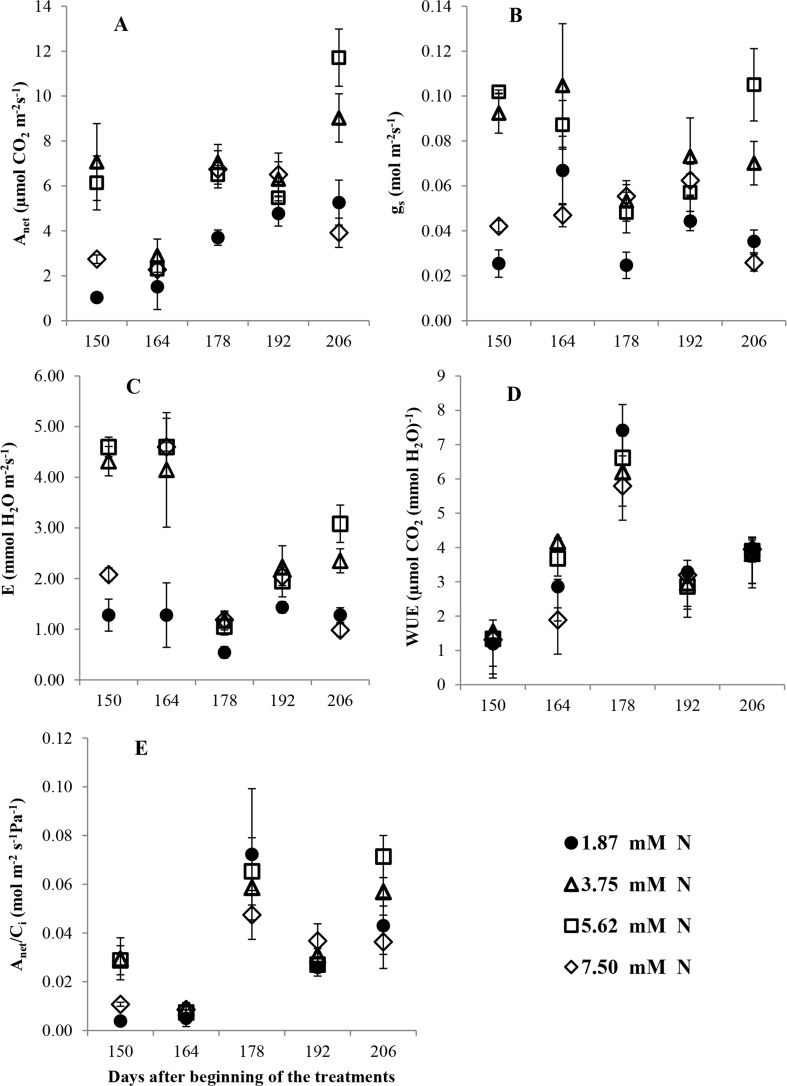
(A) Net assimilation rate (*A*_*net*_, μmol CO_2_ m^−2^ s^−1^); (B) stomatal conductance (*g*_*s*_, mol m^−2^ s^−1^); (C) transpiration (*E*, mmol water vapor m^−2^ s^−1^); (D) water-use efficiency [*WUE*, μmol CO_2_ (mmol H_2_O^−1^)]; and (E) apparent carboxylation efficiency (*A*_*net*_/*C*_*i*_, mol m^−2^ s^−1^ Pa^−1^) in *Annona emarginata* subjected to 1.87, 3.75, 5.62 and 7.5 mM N at 150, 164, 178 and 206 days after beginning treatment (DBT). Data are presented as the mean ± SE (*n* = 4).

The plants subjected to intermediate N levels (5.62 and 3.75 mM) showed a high CO_2_ assimilation rate (*A*_*net*_), stomatal conductance (*g*_*s*_), transpiration rate (*E*), and carboxylation efficiency (*A*_*net*_/*C*_*i*_) throughout the experimental period (Fig 1). These plants also showed a high maximum carboxylation velocity (*V*_*cmax*_) and respiratory rate (*R*_d_*) at 192 DBT (Table 1). For the plants grown with 7.5 mM N, the values of V_*cmax*_, *J*, and R_d_* were high (Fig 1 and Table 1).

**Table 1:** Photosynthetic potential of *Annona emarginata* grown under different nitrogen concentrations at 192 days after beginning treatment.

Nitrogen Concentration			
	V_*cmax*_	*J*	Rd*
1.87 mM	74.00 ± 15.52 b	98.33 ± 19.50	2.57 ± 0.99 b
3.75 mM	103.67 ± 22.01 ab	85.67 ± 11.85	2.93 ± 0.71 b
5.62 mM	129.67 ± 17.50 a	106.33 ± 9.50	4.96 ± 0.12 a
7.50 mM	134.67 ± 1.53 a	102.33 ± 0.58	5.19 ± 0.72 a

Data are presented as the mean ± SE (*n* = 3).

### Net assimilation and relative growth rates

**Fig 2:**
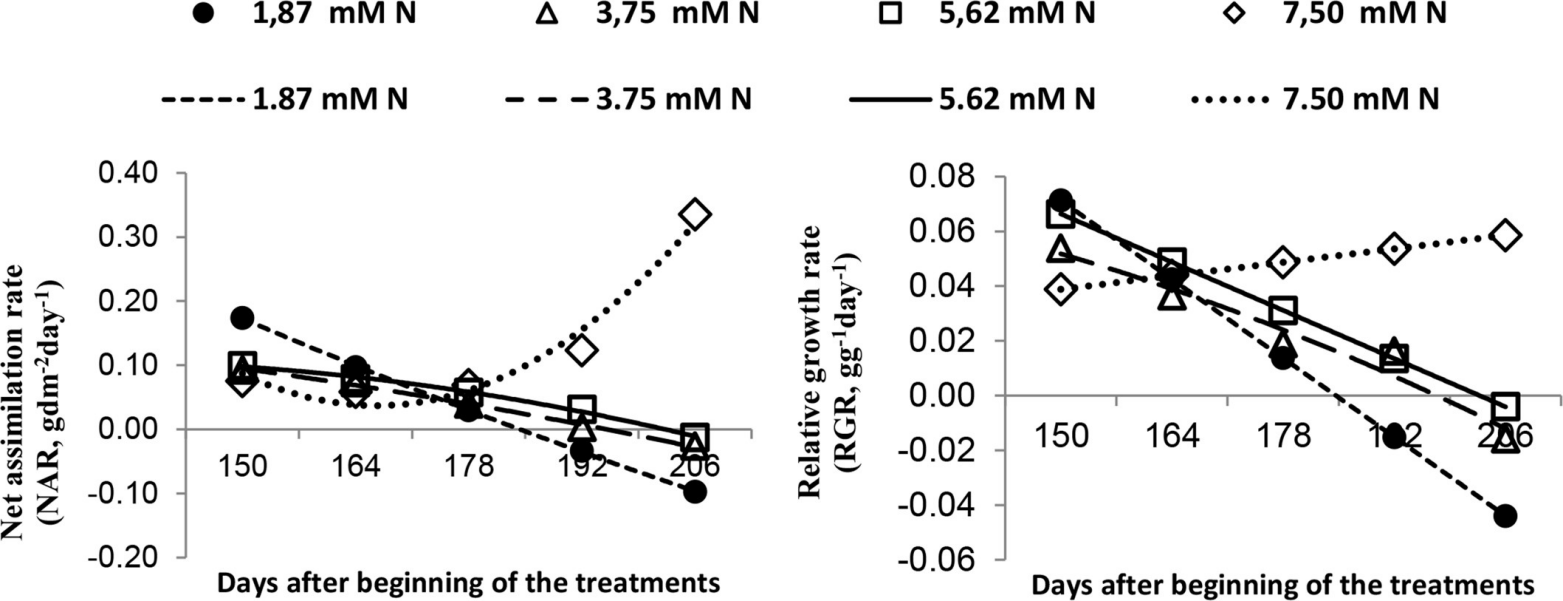
(A) Net assimilation rates (NAR, dm^2^ g); (B) relative growth rates (RGR, g g^−1^ day^-1^); and (C) leaf-specific weight (LSW, dm^2^ g^−1^) of *Annona emarginata* subjected to 1.87, 3.75, 5.62 and 7.5 mM N at 150, 164, 178 and 206 days after beginning treatment (DBT). S1 Fig.

The net assimilation and relative growth rates decreased over time in the plants grown with 1.87, 3.75, and 5.62 mM N but increased in those grown with 7.5 mM N (Fig 2). The leaf-specific weight varied slightly among the different treatments and increased from 192 DBT onward in the plants grown with 7.5 mM N (Fig 2).

### Nitrate reductase activity

**Fig 3:**
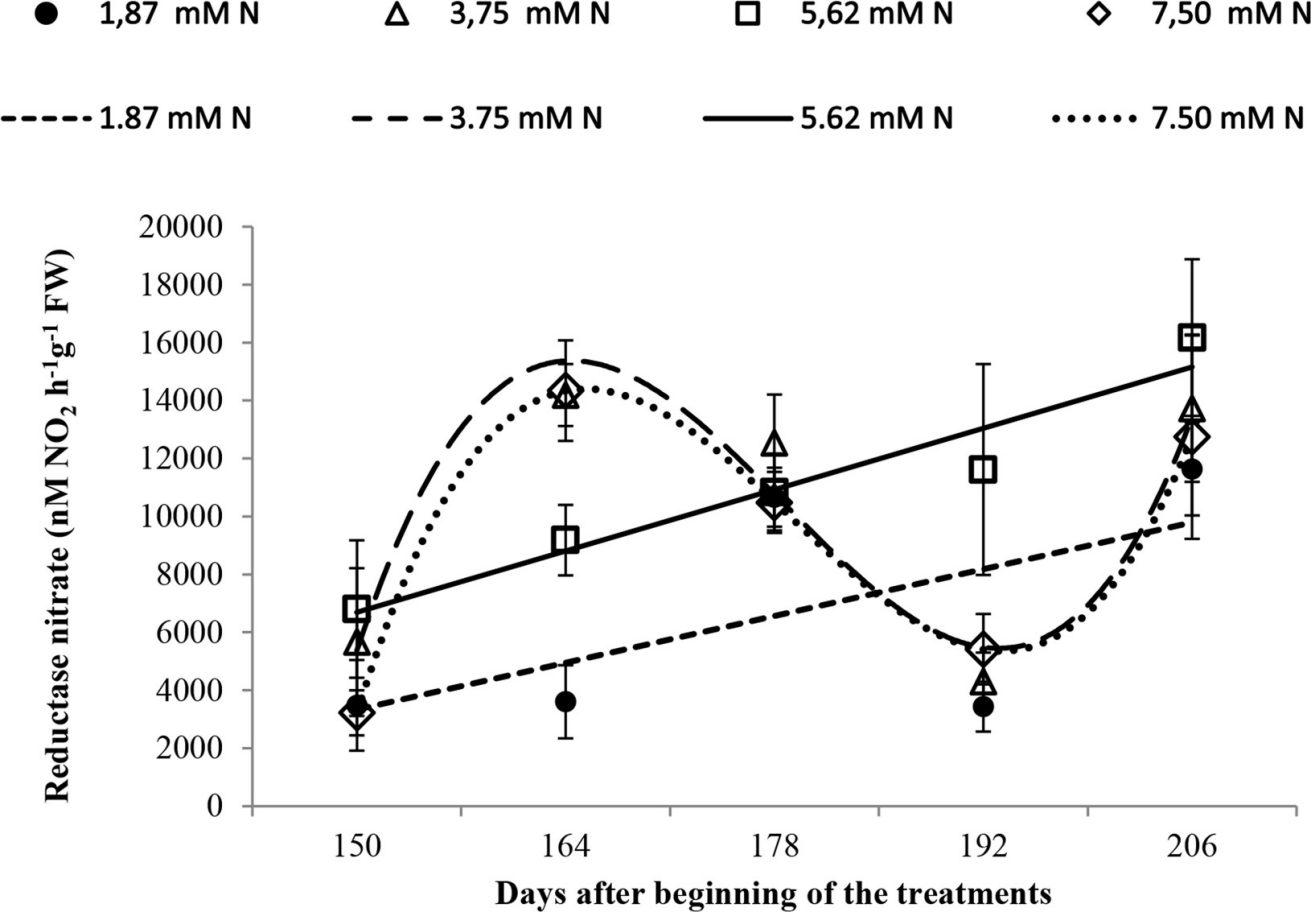
Nitrate reductase activity in *Annona emarginata* leaves subjected to 1.87, 3.75, 5.62 and 7.5 mM N at 150, 164, 178 and 206 days after beginning treatment (DBT). Data are presented as the mean ± SE (*n* = 4). S2 Fig.

The activity of nitrate reductase increased over time in plants grown with 1.87 mM N but with less activity (*p* ≤ 0.05) at 164 and 192 DBT (Fig 3).

The plants grown with 3.75 and 7.5 mM N showed an increase in nitrate reductase enzyme activity until 164 DBT (Fig 3), whereas the plants grown with 5.62 mM N showed an increase in nitrate reductase activity over time, with increased activity at 192 and 206 DBT (*p* ≤ 0.05; Fig 3).

### Leaf total amino acid concentration

**Fig 4:**
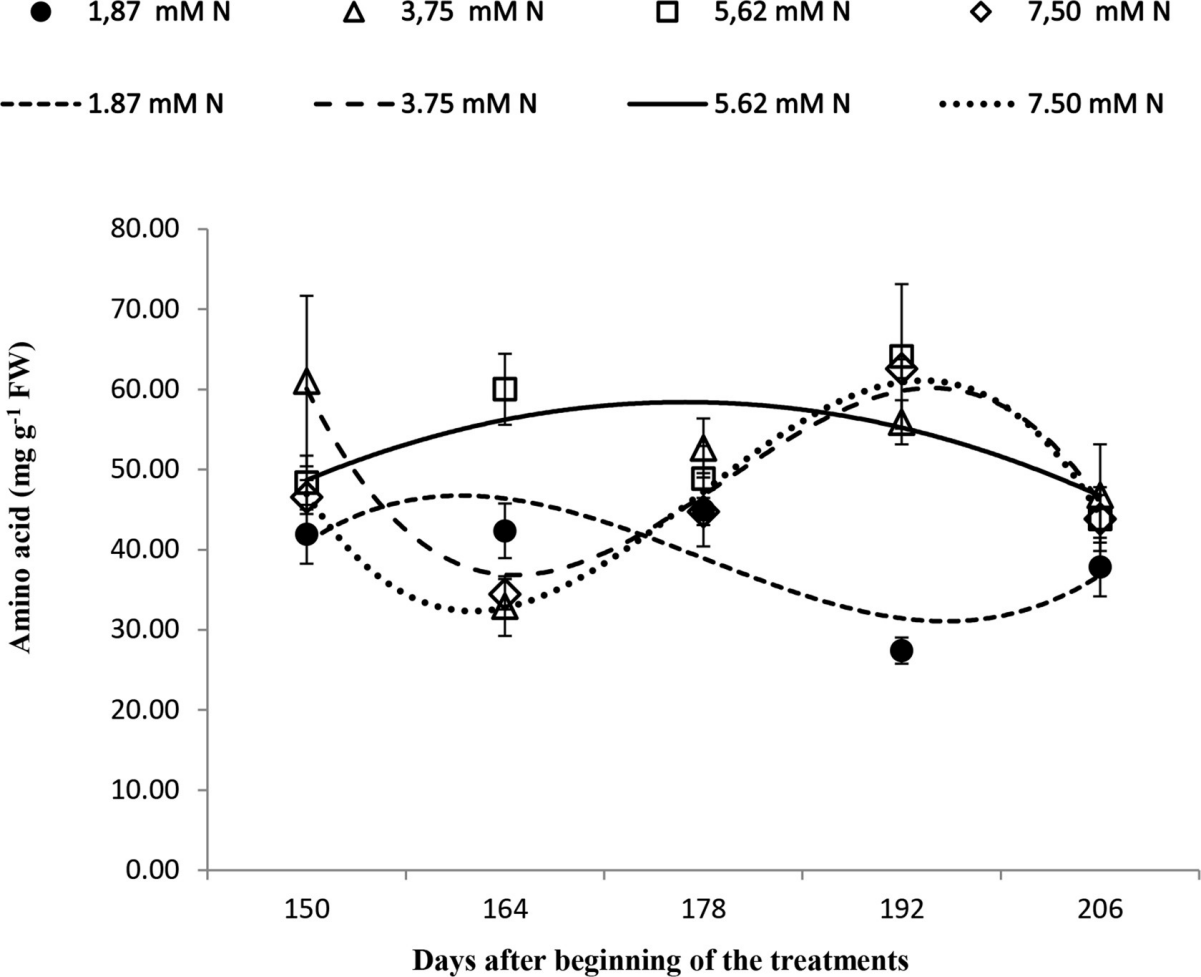
Leaf amino acid concentration of *Annona emarginata* subjected to 1.87, 3.75, 5.62 and 7.5 mM N at 150, 164, 178 and 206 days after beginning treatment (DBT). Data are presented as the mean ± SE (*n* = 4). S3 Fig.

Overall, for the plants grown with 1.87 mM N, the amino acid content decreased over time (Fig 4), whereas the plants grown with 3.75, 5.62, and 7.5 mM N showed an increase at 192 DBT (*p* ≤ 0.05).

### Leaf carbohydrate concentration

**Fig 5:**
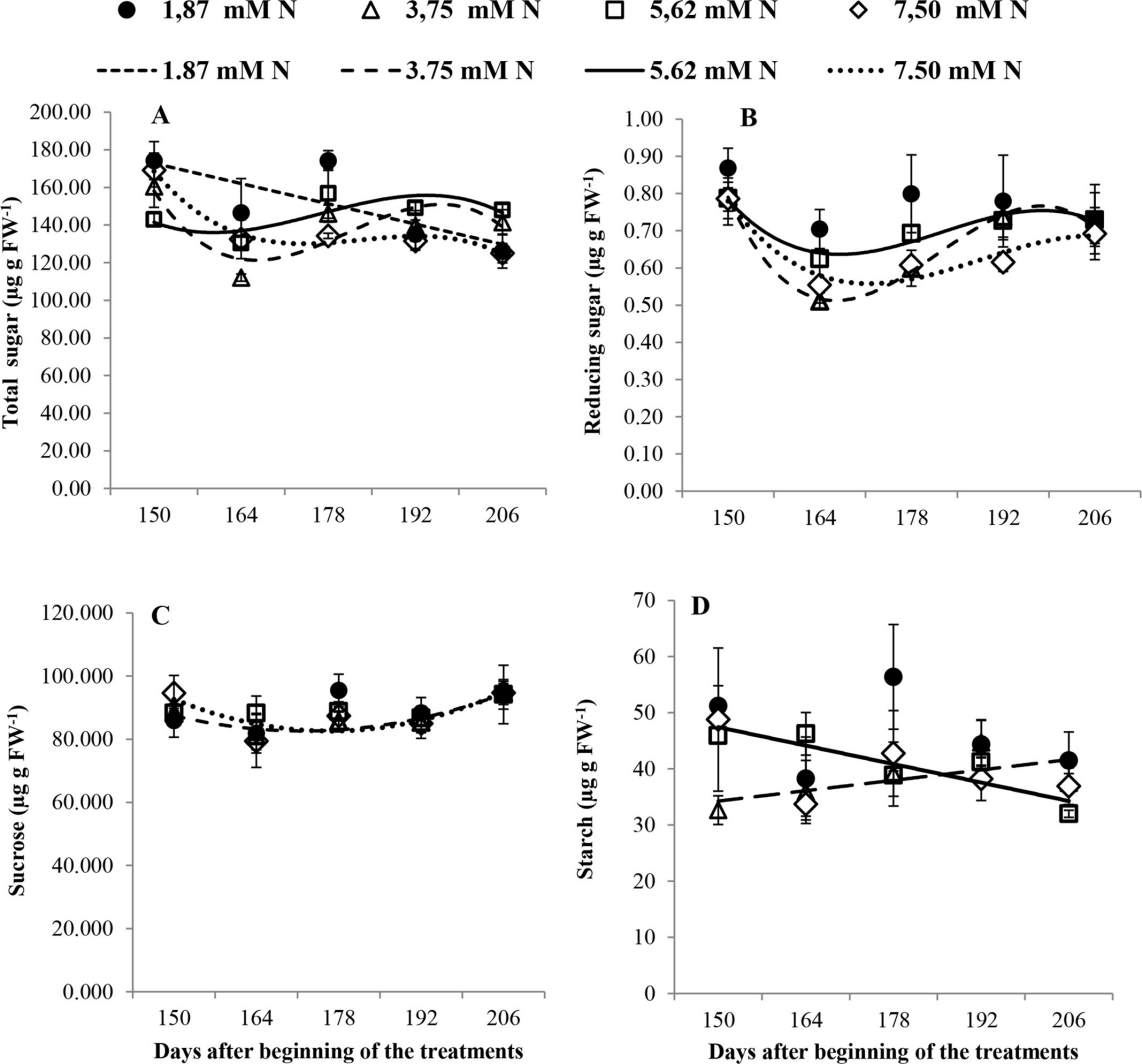
(A) Total sugar (μg g FW^−1^); (B) reducing sugar (μg g FW^−1^); (C) sucrose (μg g FW^−1^); and (D) starch (μg g FW^−1^) in *Annona emarginata* subjected to 1.87, 3.75, 5.62 and 7.5 mM N at 150, 164, 178 and 206 days after beginning treatment (DBT). Data are presented as the mean ± SE (*n* = 4). **S4 Fig**.

Generally, the plants grown with 1.87 mM N presented the highest total soluble sugar concentration (*p* ≤ 0.05), which decreased over time, and showed high and constant values of starch and reducing sugars (Fig 5).

The plants grown with 3.75 mM N presented low concentrations of total and reducing sugars at 164 and 178 DBT (*p* ≤ 0.05). The starch concentration increased over time but with reduced concentrations at 150 and 178 DBT (*p* ≤ 0.05; Fig 5). The plants grown with 7.5 mM N showed low accumulation of total and reducing sugars at 164 and 178 DBT (*p* ≤ 0.05) and a constant concentration of starch (Fig 5).

Although the carbohydrate concentration of plants grown with 5.62 mM N was relatively stable over time (Fig 5), the starch concentration decreased at 178 DBT (Fig 5).

The sucrose concentration remained constant over time and did not differ among the treatments (Fig 5).

### The activity of antioxidant enzymes and lipid peroxidation

**Fig 6:**
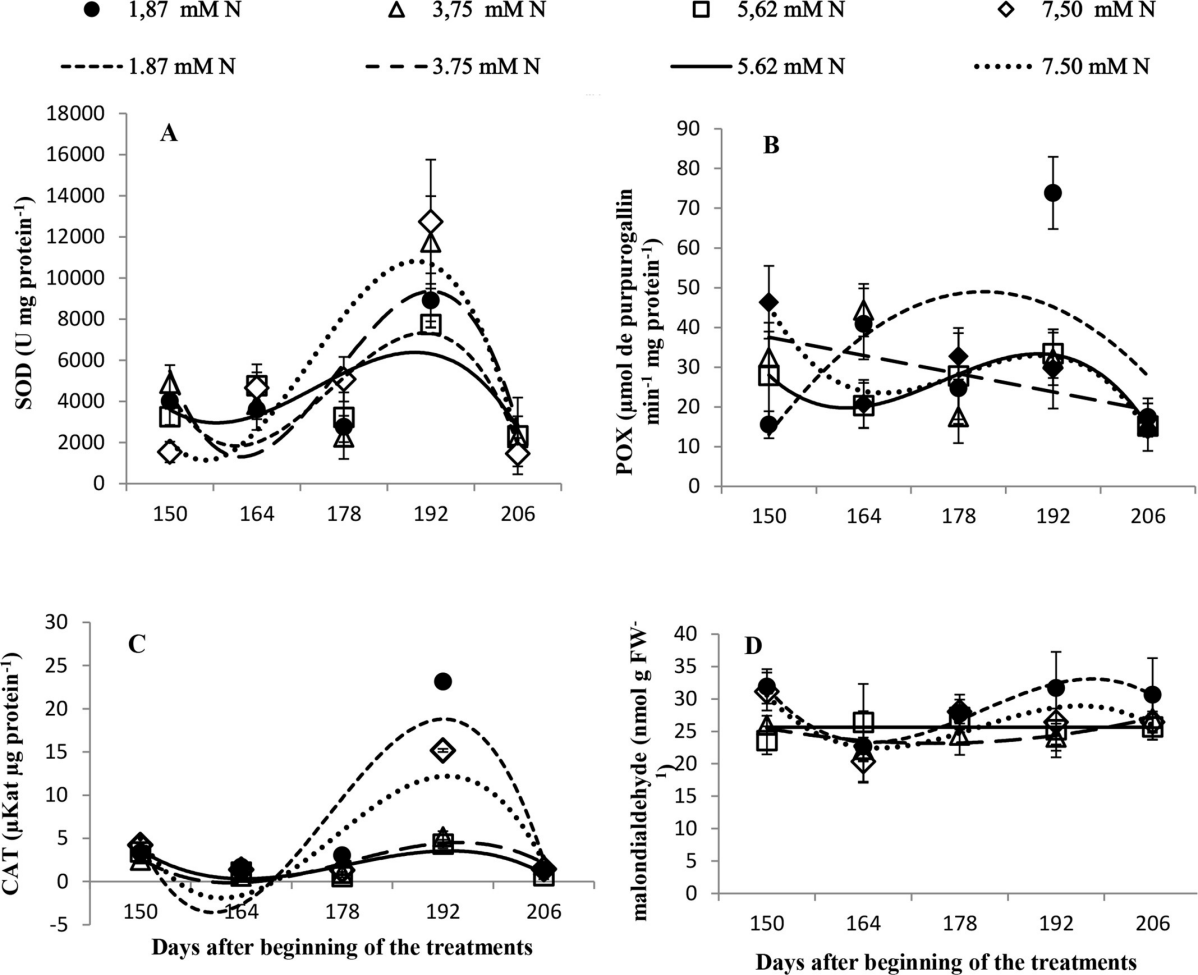
**(**A) Lipid peroxidation (MDA, malondialdehyde nmol g^−1^ of FW); (B) superoxide dismutase (SOD, U mg^−1^ protein); (C) peroxidase (POX, **μ**mol of purpurogallin min^−1^ mg^−1^ protein); and (D) catalase (CAT, **μ**Kat **μ**g^−1^ protein) of *Annona emarginata* subjected to 1.87, 3.75, 5.62 and 7.5 mM N at 150, 164, 178 and 206 days after beginning treatment (DBT). S5 Fig.

In general, SOD activity increased at 192 DBT in all plants, regardless of the nitrate level. The highest activity was in the plants grown with 7.5 mM N, compared with the activity in those grown with 5.62 mM N (*p* ≤ 0.05; Fig 6).

The plants grown with 1.87 mM N showed the highest POX activity at 192 DBT (*p* ≤ 0.05; Fig 6).

CAT activity was the highest in plants grown with 1.87 mM N at 192 DBT and the lowest in plants grown with 5.62 mM N (*p* ≤ 0.05; Fig 6). Lipid peroxidation did not change with the different nitrate levels (*p* ≤ 0.05; Fig 6).

### Analysis and identification of volatile substances

Forty-eight substances were identified in the profile of volatiles from the leaves of *A*. *emarginata* cultivated at varying nitrogen concentrations (Table 2). Volatiles were classified as signaling and defense substances (mono- and sesquiterpenes). Signaling substances were the volatile substances with the lowest molecular mass detected in the leaves of *A*. *emarginata*. The relative abundances of these volatiles were low, but they increased when the nitrate concentration decreased (F = 18.117, *p* ≤ 0.001). In general, in the high-nitrate treatments (7.5 and 5.62 mM N), the sum of the values was close to 0.5 and 1.5%, respectively, and in the low-nitrate treatments (1.87 and 3.75 mM N), these leaf volatiles reached 3% relative abundance (Table 2).

**Table 2:** Mean relative percentages and number of volatiles of *A*. *emarginata* subjected to 1.87, 3.75, 5.62 and 7.5 mM N at 150, 164, 178 and 206 days after beginning treatment (DBT).

	**Nitrogen concentration (mM)**																			
	**7.5**					**5.62**					**3.75**					**1.87**				
	**150**	**164**	**178**	**192**	**206**	**150**	**164**	**178**	**192**	**206**	**150**	**164**	**178**	**192**	**206**	**150**	**164**	**178**	**192**	**206**
**Signaling substances**	**Days after beginning the treatments (DBT)**																			
**Heptanal**	0bB	0bB	0bC	0.25aA	0bC	0aB	0aB	0aC	0aB	0aC	0bB	0bB	0.34 aB	0.27aA	0.28aA	0.39aA	0.35aA	0.43aA	0cB	0.19bB
**n-nonanal**	0bC	0bB	0bC	0.26aB	0bB	0.40bB	0.81 aA	0.65 abB	0.18 cB	0.47bA	0.51 bAB	0.67 abA	0.49 bB	0.72aA	0.64 abA	0.62bA	0.69bA	1.15 aA	0.29cB	0.45 bcA
**n-decanal**	0aB	0aB	0aC	0aB	0aB	0aB	0aB	0aC	0aB	0aB	0bB	0bB	0.24aB	0.25aA	0.17aA	0.18bA	0.23bA	0.87aA	0.28bA	0.25bA
**6-methyl-5-hepten-2-one**	0aB	0aB	0aC	0aB	0aC	0cB	0.27 abA	0.31aB	0.00 cB	0.17bB	0.55 aA	0cB	0.33 bB	0.25bA	0.30bA	0cB	0.29bA	0.52 aA	0.35bA	0.30bA
**hexyl acetate**	0bC	0bB	0.46 aAB	0bB	0bC	0.27aB	0.41 aA	0.25 abB	0.09 bB	0.27aB	0.12 bBC	0.32aA	0.46aA	0.32aA	0.48aA	0.59aA	0.29bA	0.30 bB	0.25bA	0.43 abAB
**%**	**0**	**0**	**0.46**	**0.51**	**0**	**0.67**	**1.48**	**1.21**	**0.26**	**0.91**	**1.17**	**0.98**	**1.86**	**1.8**	**1.86**	**1.77**	**1.84**	**3.26**	**1.16**	**1.61**
**N°**	**0**	**0**	**1**	**2**	**0**	**2**	**3**	**3**	**2**	**3**	**3**	**2**	**5**	**5**	**5**	**4**	**5**	**5**	**4**	**5**
**Defense substances Monoterpene**																				
**α-tujene**	12.78aA	3.80bA	10.21 aA	4.29 bBC	2.40bB	7.75 abB	5.15 bA	1.96cC	9.18 aA	7.62 abA	7.57 aB	6.24aA	6.57 aB	6.17aB	1.83bB	11.88 aA	4.44cA	5.54 bcB	3.07cC	8.18bA
**α-pinene**	3.63aA	1.46bA	3.73aA	1.60bA	0.99bA	2.96aA	2.68 aA	1.86aB	2.40 aA	2.42aA	2.15 aA	2.10aA	1.82 aB	2.67aA	1.78aA	3.94aA	1.17bA	1.06 bB	1.30bA	2.44 abA
**Sabinense**	0bC	0bC	0bB	0bB	0.89 aB	1.41 abB	1.96aA	1.04bA	1.52 abA	1.68 abA	1.45 aB	1.22 aAB	1.17 aA	1.53aA	0.77aB	2.52aA	0.90bB	1.06 bA	0.77 bAB	1.72 abA
**β-pinene**	0.74bA	0.69bA	1.75aA	0.66bA	0.33 cA	0.23 abB	0bB	0bB	0.22bB	0.39aA	0aB	0.32aB	0.27aB	0.25aB	0.35aA	0.29 abB	0bB	0bB	0.45 aAB	0.19 abA
**Mircene**	0cC	0.33bB	0.69aA	0.40bB	0cC	0.27bB	0.39 bB	0.68aA	0.88 aA	0.82aB	0bC	0.69aA	0.72 aA	0.67aA	0.75a	0.74bA	0.67bA	0.65 bA	0.54bB	1.42aA
**δ-3-carene**	0aB	0aC	0aB	0aC	0aC	0bB	0bC	0bB	0.38aA	0.35aB	0cB	0.34bA	0.43aA	0.26bB	0cC	0.20bA	0.24bB	0cB	0.26bB	0.55aA
**o-cimene**	0bB	0bB	0bC	0.22aA	0bC	0bB	0bB	0.26aB	0.16 abA	0.26aB	0.50 aA	0.41 abA	0.32 abB	0.20bA	0.40 abB	0.67aA	0bB	0.54 aA	0.29bA	0.70aA
**Limonene**	0aC	0aC	0aB	0.10aC	0aC	0.24aB	0bC	0.42aA	0.35 aB	0.40aB	0cC	0.63aA	0.39 bAB	0.67aA	0.41bB	0.46bA	0.31bB	0.41 bA	0.66aA	0.80aA
**1,8-cineol**	0aA	0aB	0a	0a	0a	0aA	0aB	0aA	0aB	0aB	0cA	0.25bA	0cA	0.25bA	0.90aA	0aA	0aB	0aA	0aB	0aB
***Trans*-β-ocimene**	0aA	0aA	0aA	0aB	0aB	0aA	0aA	0aA	0aB	0aB	0bA	0bA	0bA	0bB	0.18aA	0bA	0bA	0bA	0.18aA	0bB
**γ-terpinene**	0aB	0aC	0aC	0aB	0aB	0bB	0bC	0.22aB	0.31 aA	0.17aA	0cB	0.39aA	0.43 aA	0cB	0.17bA	0.65aA	0.21bB	0.23 bB	0.22 bAB	0.19bA
**terpinen-4-ol**	0aB	0aA	0aC	0aB	0aB	0bB	0bA	0.13aB	0bB	0bB	0cB	0cA	0.29 aA	0.19bA	0cB	0.25aA	0bA	0bC	0bB	0.22aA
**%**	**17.15**	**6.27**	**16.37**	**7.25**	**4.61**	**12.85**	**10.17**	**6.56**	**15.38**	**13.71**	**11.67**	**12.58**	**12.39**	**12.84**	**7.52**	**21.57**	**7.93**	**9.48**	**7.72**	**16.39**
**N°**	**3**	**4**	**4**	**6**	**4**	**6**	**4**	**8**	**9**	**8**	**4**	**10**	**10**	**10**	**10**	**10**	**7**	**7**	**10**	**10**
**Defense substances Sesquiterpene**																				
**δ-elemene**	0cA	0.98bA	0.32cA	2.55aA	0.55 bcB	0.12aA	0.31 aB	0.51aA	0.39 aB	0.55aB	0.30bA	0.68bAB	0.34bA	0.34bB	1.89aA	0.29aA	0.10aB	0.38aA	0.26aB	0.27aB
**α-cubebene**	0cB	0cB	0.51 abA	0.62aA	0.39bA	0.31bA	0cB	0.57aA	0.34 bB	0.33bA	0.24 aA	0.30aA	0.26 aB	0.37aB	0bB	0.19aA	0.28aA	0.28 aB	0bC	0.32aA
**Cyclosativene**	0aB	0aB	0aC	0aB	0aB	0bB	0bB	0.25aA	0bB	0bB	0cB	0.09bA	0cC	0.17abA	0.24aA	0.15bA	0cB	0.09bcB	0cB	0.29aA
**Longicyclene**	0aA	0aB	0aA	0aA	0aB	0aA	0aB	0aA	0aA	0aB	0bA	0.09aA	0bA	0bA	0bB	0bA	0bB	0bA	0bA	0.19aA
**α-copaene**	0.89bB	1.51bBC	2.77aA	1.38 bAB	2.67aB	2.32bA	1.62 bcB	3.44aA	1.65bcA	0.95cC	1.37 cB	2.88bA	0.86 cB	1.08 cAB	5.83aA	1.79 aAB	0.68bC	1.29 abB	0.80bB	1.52aC
**β-bourbonene**	0.98bcA	0.40cAB	0.70cC	1.49bB	3.69aA	0.89cA	0.24dB	2.74aA	2.30abA	1.94bC	1.23bA	0.46cAB	0.40cC	1.20bBC	1.98aC	0.33dB	0.90bcA	1.45bB	0.65cdC	2.74aB
**β-cubebene**	0.82bAB	0.81bA	2.22aA	1.20 bAB	1.26bA	0.94 bAB	0.21 cB	1.62aB	1.47 abA	1.35 abA	1.28 aA	0bB	0.48 bC	0.76 abB	1.17aA	0.48bB	1.09aA	1.51 aB	0bC	1.43aA
**β-elemene**	7.63aA	7.56 aA	1.37cA	1.09cB	4.48bB	7.85aA	0.85 cC	0.84cA	4.57 bAB	5.34 bAB	3.34 bB	2.04 bcBC	1.05 cA	1.72bB	7.26aA	0.98cC	3.69bB	0.68 cA	4.84 abA	6.78aA
**Ciperene**	0cC	0.80aA	1.11aA	0.42bA	0cB	1.15aA	0.55bA	0.70bB	0cB	0.71bA	0.90aAB	0.70abA	0.37bC	0.68abA	0.07bB	0.67aB	0.47aA	0.60aB	0.39aA	0bB
***cis*-caryophyllene**	1.95aC	0.63bA	0bA	0bA	0bA	5.47aA	0.56 bA	0bA	0.35 bA	0bA	5.44 aA	0.27bA	0.13 bA	0.19bA	0bA	3.57aB	0.22bA	0bA	0bA	0bA
**α-gurjunene**	0aB	0aB	0aB	0aB	0aB	0aA	0aA	0aA	0aA	0aB	0aA	0aA	0aA	0aA	0aB	0bA	0bA	0bA	0bA	0.15aA
***trans*-caryophyllene**	34.26aB	19.84bcB	21.36 bB	12.22 cB	25.47 bA	26.70 aC	28.69 aA	15.17 bB	24.78 aA	10.53 bB	43.62 aA	30.48 bA	29.83 bA	9.58cB	17.23 cB	38.93 aAB	24.21 bAB	21.45 bB	24.74 bA	12.05 cB
**β-copaene**	0.55aAB	0.43 aA	0.48aB	0.69aA	0.65aB	0.32bB	0.52 bA	0.85aA	0.76 abA	0.95aA	0.60 abA	0.49 abA	0.43 bB	0.75aA	0.67 abB	0.34bB	0.47bA	0.53 bB	0.40bB	0.82 aAB
**β-gurjunene**	0cA	0.34bA	0cC	0.39 abA	0.51aA	0cA	0cB	0.45aA	0.27 bAB	0.47aA	0bA	0.27aA	0.16 abB	0.27 aAB	0bC	0bA	0.39aA	0.28 aB	0.23aB	0.30aB
**aromadendrene**	0.77aA	0.56 aA	0.79 aAB	1.13aA	0.61aA	0.87aA	0.59 aA	0.42aB	0.50 aB	0.84aA	0.51 aA	0.39aA	0.51 aAB	0.67aB	0.50aA	0.67 abA	0.73 abA	2.24 aA	0.48bB	0.35bA
**α-humulene**	3.33aA	2.12bB	2.19bA	1.84bA	2.36 abAB	3.75aA	3.65 aA	3.07 abA	2.45 bcA	1.54cB	4.04 aA	3.57 abAB	2.94 bA	1.77cA	3.05bA	3.28aA	2.57 abB	2.15 bA	2.51 abA	1.69bB
**allo-aromadendrene**	1.26aB	1.53aA	1.59aA	1.29aA	0.69bA	1.82aA	1.06 bAB	0.95bcB	1.30bA	0.54cA	0.67 aC	0.93aC	1.09 aB	0.66aB	0.66aA	1.30aB	1.39aB	0.39 bC	0.47bB	0.65bA
***cis*-cadine-1 (6),4 diene**	0cB	0.63bB	0.92aB	1.06aA	0.71bA	0.09cB	0cC	1.22aA	0.58 bB	0.74bA	0.70 aA	0.78 aAB	0.45 bC	0.44 bBC	0.72aA	0cB	0.83aA	0.38 bC	0.37bC	0.81aA
***cis*-muurole-4 (14),5 diene**	0aA	0aA	0aA	0aB	0aC	0cA	0cA	0cA	0.17 bA	0.37aA	0bA	0bA	0bA	0.21aA	0.22aB	0bA	0bA	0bA	0bB	0.33aA
**γ-gurjunene**	2.28cB	2.90 cC	22.55 aA	21.53 aA	11.27 bB	1.04 cdB	1.46 cC	21.07 aA	13.82 bB	13.92 bB	7.65 bcA	11.91 abB	6.44 bcB	6.12cC	16.09 aAB	1.27cB	16.75 aA	12.19 bB	6.03cC	19.73 aA
***trans*-muurole-4 (14),5 diene**	0dB	0.38bA	0dB	0.52aA	0.29 cB	0.09cA	0dB	0.48aA	0.27 bC	0.42aA	0aB	0aB	0aB	0aD	0aC	0cB	0.39aA	0cB	0.34bB	0aC
**bicyclogermacrene**	15.08 aA	16.07 aB	19.61 aA	22.12aAB	16.83aA	4.34cB	15.34bB	19.48abA	24.86aA	22.14abA	6.81bB	16.85aB	17.20aA	20.38aAB	15.76aA	9.24cAB	27.29aA	17.93bA	15.22bcB	15.15bcA
***trans*-β-guaiene**	0.94aA	0.89 abA	0.75abA	0.63bB	0.73 abB	0.70 abAB	0.61bB	0.89 abA	0.	0.63bB	0.79aAB	0.69 aAB	0.69aA	0.80aB	0.70aB	0.66bB	0.46bB	0.64bA	1.18aA	1.46 aA
**germacrene A**	1.90abAB	2.41aB	0.67bA	1.17bB	2.72 aA	1.95aA	0.55bC	0.38bA	1.14 abB	1.83 aA	0.73bB	1.48 abBC	0.72bA	0.66bB	2.06aA	2.13bA	3.75aA	0.08cA	2.52bA	2.52 bA
**γ-cadinene**	1.10aA	1.11aA	0.40bB	0.93aA	0bB	0.51bB	0.44bB	1.18aA	0.66bA	1.19a	0.95aA	0.69aB	0.71aB	0.96aA	1.04a	0.44cB	0.62 bcB	1.54aA	0.65 bcA	0.95b
**δ-cadinene**	1.31 bcA	1.46bA	0.77cB	2.37aA	0.70cC	1.47 abA	1.39 abA	1.98aA	0.96bB	1.51 abB	1.40cA	1.13cA	1.04cB	2.27bA	3.56aA	1.63aA	1.05 abA	0.93bB	1.20 abB	1.02 abBC
**α-cadinene**	0aA	0aA	0aB	0aA	0aB	0bA	0bA	0.22aA	0bA	0bB	0bA	0bA	0bB	0bA	0.14aA	0aA	0aA	0aB	0aA	0aB
**germacrene B**	0.12 bAB	0.16bA	0bA	0.99aA	0.44 abB	0.60 abA	0.29bA	0bA	0.28 bBC	1.10 aA	0bB	0.23bA	0.27bA	0.82 bAB	1.55aA	0.28 aAB	0aA	0aA	0.22aC	0.28aB
**Spathulenol**	0bB	0bB	0bB	0bB	0.54 aA	0.28 aAB	0.30aA	0.44aA	0.25aA	0bC	0.44aA	0.30aA	0bB	0.31aA	0bC	0.20 abB	0.22aA	0bB	0.18 abAB	0.27aB
**caryophyllene oxide**	0.82aA	0.35 bcB	0dB	0.06 cdB	0.48 bA	0.50bB	1.17aA	0.51bA	0.24 bAB	0.50bA	0.51aAB	0.31 abB	0.39 abA	0.43 abA	0.20bA	0.16aC	0.24aB	0.21aAB	0.26 aAB	0.33 aA
**epi-α-cadinol**	0bB	0.35aA	0bA	0bB	0bB	0aB	0aB	0aA	0aB	0aB	0.39aA	0cB	0cA	0.25bA	0cB	0.43bA	0cB	0.08cA	0.25bA	0.34abA
**%**	**775.95**	**664.18**	**881.05**	**777.67**	**777.98**	**664.01**	**558.74**	**779.38**	**885.26**	**770.32**	**883.87**	**777.95**	**666.76**	**553.78**	**882.55**	**669.35**	**888.77**	**667.25**	**664.14**	**772.69**
**N°**	**18**	**24**	**19**	**23**	**22**	**24**	**20**	**25**	**25**	**24**	**23**	**25**	**23**	**27**	**23**	**24**	**24**	**23**	**23**	**27**
**N° TOTAL**	**21**	**28**	**24**	**31**	**26**	**32**	**27**	**36**	**36**	**35**	**30**	**37**	**38**	**42**	**38**	**38**	**36**	**35**	**37**	**42**

The lowercase letters indicate the differences in the time of evaluation (DBT) for each nitrogen concentration (mM N), and the uppercase letters indicate the differences in the concentrations of nitrogen at the evaluation time. The means were compared using Tukey’s test, with the probability level of 5%.

Defense substance monoterpenes were the second most abundant class of volatile substances in the leaves of *A*. *emarginata*. The abundance of monoterpenes varied with nitrate concentration and time of evaluation (F = 16,308, *p* ≤ 0.001). The highest nitrate content (7.5 mM N) resulted in a low relative percentage of monoterpenes of 6.27, 7.25 and 4.61% at 164, 192 and 206 days after beginning treatment, respectively. The lowest concentration of nitrate (1.87 mM N) showed an increase in monoterpenes to 21.57 and 16.39% at 150 and 206 DBT (Table 2).

**Fig 7:**
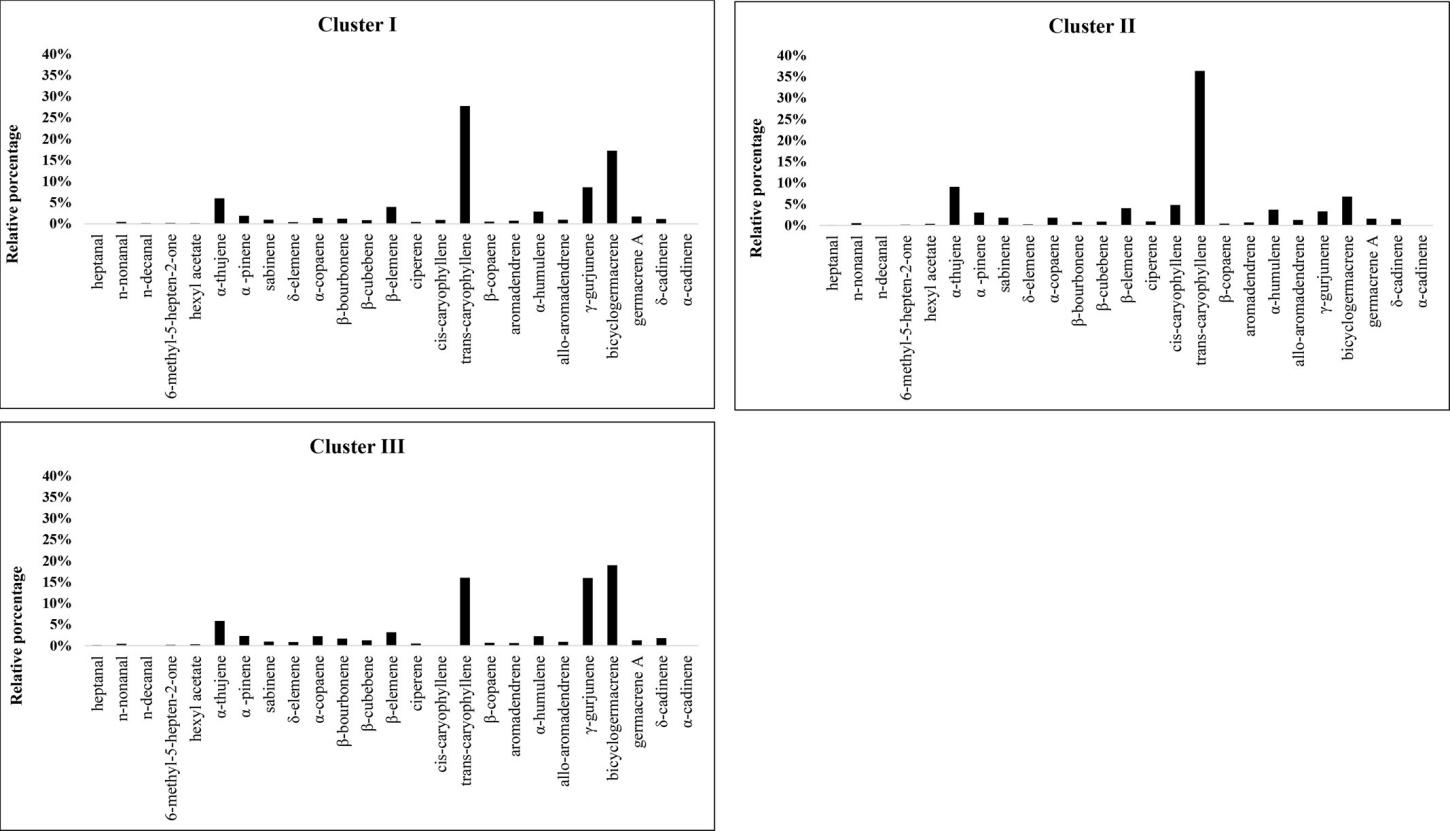
Means of the 25 most important volatile substances in Clusters I, II, and III of *A*. *emarginata*.

Defense substance sesquiterpenes contained the most substances (up to 31) and were the most abundant class (approximately 80%). The density of the sesquiterpene class was also influenced by nitrate concentration and time of evaluation (F = 22.670, p ≤ 0.001; Table 2). The most frequent and abundant sesquiterpenes were *trans*-caryophyllene, bicyclogermacrene and γ-gurjunene (Table 2 and Fig 7).

The diversity indices (H) for *A*. *emarginata* with different concentrations of nitrate confirmed that the species produced greater phytochemical diversity under low concentrations of nitrate and at later evaluation times. The volatile substance indices were high in the treatments with low nitrate (3.75 and 1.87 mM), particularly at the last evaluation. At 206 days, the low nitrate treatments were 16 and 18% more diverse (H: 2.66 and 2.71, respectively) than the treatment with the highest concentration of nitrate (H: 2.3; Table 3).

**Table 3:** Diversity indices of the volatile substances (H index) of *A*. *emarginata* subjected to 1.87, 3.75, 5.62 and 7.5 mM N at 150, 164, 178 and 206 days after beginning treatment.

Nitrogen (mM)	Days after beginning of the treatments					
	150	164	178	192	206	
**7.50**	2.14 ± 0.03 Dc	2.41 ± 0.07 Aa	2.25 ± 0.04 Cb	2.38 ± 0.05 ABb	2.30 ±0.01 BCc	F = 23.09; P<0.01
**5.62**	2.48 ± 0.04 Ba	2.12 ± 0.05 Dc	2.50 ± 0.05 Ba	2.37 ± 0.03 Cb	2.61 ± 0.04 Ab	F = 74.35; P<0.01
**3.75**	2.22 ± 0.06 Cc	2.34 ± 0.05 Bab	2.25 ± 0.07 BCb	2.71 ± 0.03 Aa	2.66 ± 0.02 Aa	F = 85.24; P<0.01
**1.87**	2.31 ± 0.08 Bb	2.28 ± 0.03 Bb	2.40 ± 0.1 Ba	2.38 ± 0.01 Bb	2.71 ± 0.01 Aa	F = 30.32; P<0.01
	F = 24.63; P<0.01	F = 23.83; P<0.01	F = 11.94; P<0.01	F = 109.1; P<0.01	F = 236.2; P<0.01	

The diversity indices (H index) were subjected to analysis of variance, and the means were compared using Tukey’s test, with the probability level of 5%.

Capital letters show the differences in the row, and small letters show the differences in the columns.

**Fig 8:**
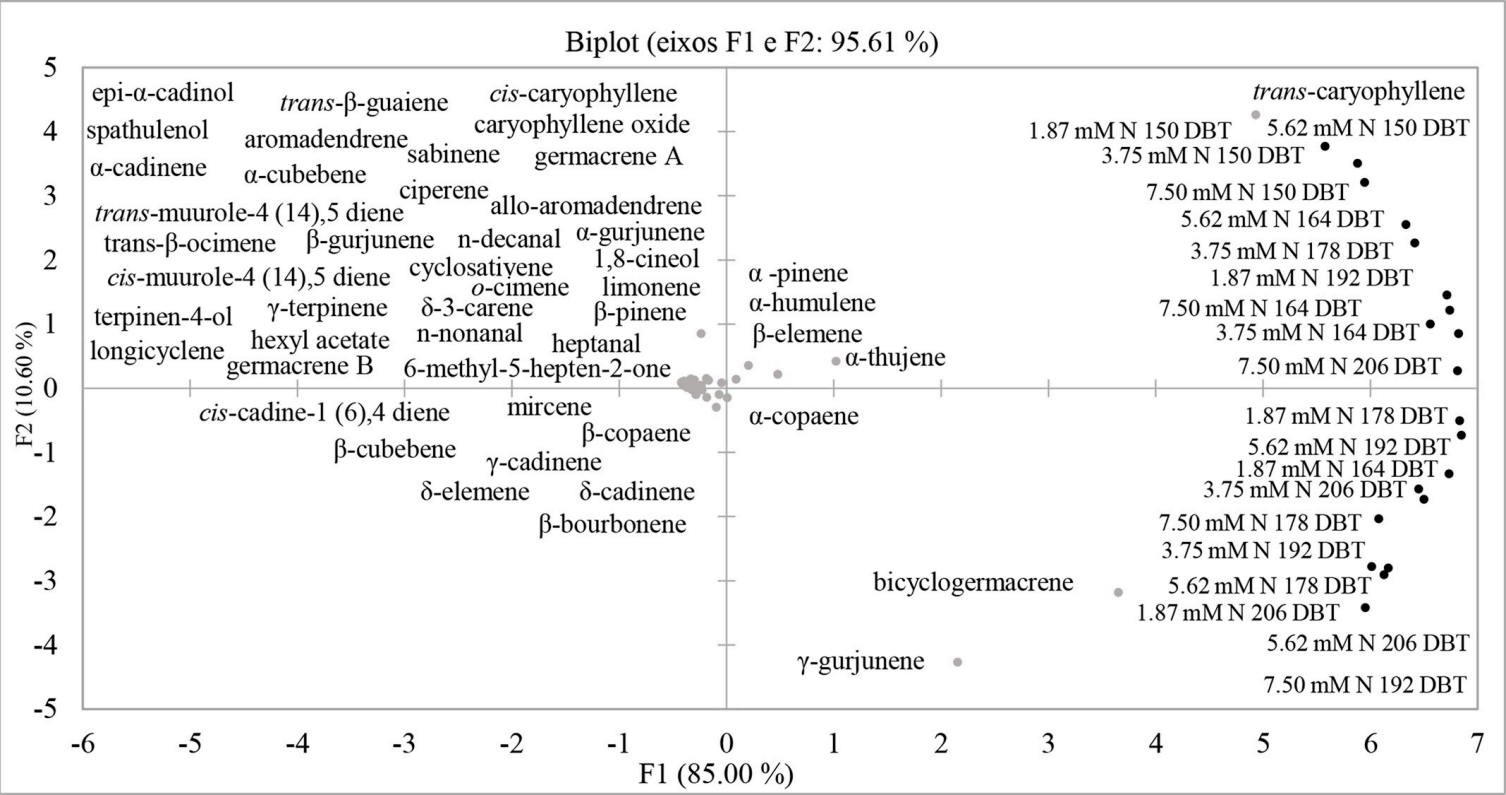
PCA of the total volatile substances with relative percentages of *A*. *emarginata* subjected to 1.87, 3.75, 5.62 and 7.5 mM N at 150, 164, 178 and 206 days after beginning treatment (DBT).

The principal component analysis (PCA) of volatile substances of *A*. *emarginata* grown at different concentrations of nitrate and evaluated at different times after beginning the treatments explained 95.61% of the chemical variation. Three primary substances were responsible for discriminating among nitrate concentrations and time points: *trans*-caryophyllene, γ-gurjunene, and bicyclogermacrene (Fig 8).

**Fig 9:**
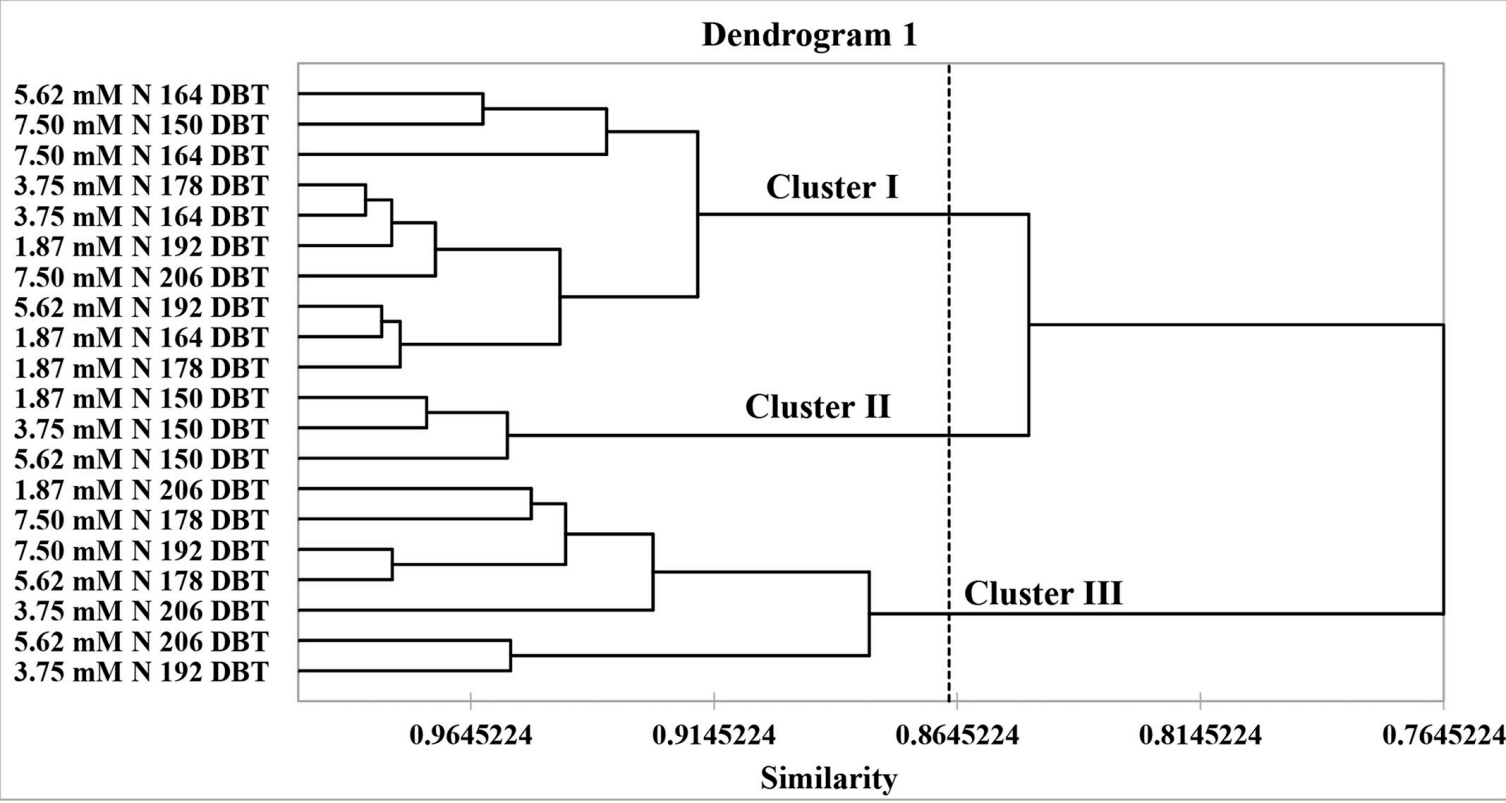
Dendrogram 1 of the volatile substances with relative percentages of *A*. *emarginata* subjected to 1.87, 3.75, 5.62 and 7.5 mM N at 150, 164, 178 and 206 days after beginning treatment (DBT).

The grouping analysis of the primary volatile substances in *A*. *emarginata* resulted in the formation of three clusters. Cluster I grouped all nitrate concentrations at 164 DBT with other time points. Cluster II constituted the three lowest nitrate concentrations at the first evaluation time point. Cluster III grouped all the concentrations and the three latest evaluation times (Figs 8 and 9). Twenty-five primary substances were responsible for these groupings and are shown in Fig 7.

## Discussion

The use of nitrate as a source of nitrogen variation in *A*. *emarginata* caused changes in the leaf volatile profile as a consequence of changes observed in the primary metabolism, which contributed to the species' defense system.

The high activity of nitrate reductase observed in *A*. *emarginata* plants grown with 5.62 mM N with their photosynthetic potential determined by the *A*_*net*_/C_i_ curve at 192 DBT helped to explain the high respiratory rate (Rd *), which might require nicotinamide adenine dinucleotide (NADH+H^+^) for nitrate reduction [55–58]. The plants cultivated at 5.62 mM N, presented constant activity of the antioxidant enzymes SOD, POX, and CAT and volatile signaling (n-nonanal, 6-methyl-5-hepten-2-one, hexyl acetate) and defense (mono- and sesquiterpenes) substances, whose action in the control of lipid peroxidation may have prevented membrane damage and allowed photosynthetic acclimatization. This would be revealed by the rapid increase in carbohydrate and amino acid concentrations as a consequence of the efficient use of reducing agents and carbon skeletons in the metabolism of carbon and nitrogen in *A*. *emarginata*. Thus, the availability of nitrogen at 5.62 mM N improved the resistance of *A*. *emarginata* because at this concentration, signaling substances were observed, with an increased number of monoterpenes and a high relative percentage of sesquiterpenes, which resulted in a high diversity index (H) at two time points. The volatile substances associated with the activity of the antioxidant enzymes might be related to the ability of nitrate to induce defense pathways of *A*. *emarginata* [11]. A concentration equal to 5.62 mM N can allow *A*. *emarginata* to be resistant to pests and diseases, which is the rootstock most common for *Annona x atemoya*.

The POX and CAT in *A*. *emarginata* grown with 3.75 mM N showed low activity, indicating that the increase in the relative percentage of the signaling substances optimized signaling and the response to stress [23]. Mono- and sesquiterpenes in these plants might have aided the antioxidant enzymes in the neutralization of reactive oxygen species, maintaining stable lipid peroxidation levels [20,59,60].

The plants grown with 1.87 mM N presented low nitrate reductase activity, resulting in the lowest free amino acid accumulation, high carbohydrate accumulation observed in the leaves until 178 DBT, and accelerated growth, as shown by the increase in the slopes of the NAR and RGR lines. These outcomes might be the result of the highest presence of ROS of all concentrations, as indicated by the high activity of antioxidant enzymes, particularly POX and CAT, at 192 DBT. ROS are powerful signaling molecules involved in plant growth control [12] that prevent plants from vegetating and accelerating their growth [61,62].

The low growth triggered by the low concentration of nitrogen contributed to the targeting of reducing agents and carbon skeletons to increase the volatile substances and carbohydrates, in particular, starch. The proportions of monoterpenes and signaling substances increased, as shown by the diversity index (H), contributing to the defense of *A*. *emarginata* to reverse stress (66) once levels of lipid peroxidation remained stable.

A high nitrate supply (7.5 mM N) can increase cell nitrite concentrations in *A*. *emarginata*, which induce nitrate reductase to reduce nitrite to nitric oxide using NADPH+H^+^ as an electron donor [63–65]. Nitric oxide affects several physiological processes in plants, including stomatal closure, which might explain the low CO_2_ assimilation observed. Additionally, the high nitrate reductase activity detected at 164 DBT led to increased amino acid accumulation and reduced levels of reducing sugar at 192 DBT, suggesting competition for reducing agents and carbon skeletons [4] because the resources produced were for amino acid synthesis. This is the most likely explanation for the increased physiological indices (NAR and RGR) shown by the plants grown at the highest N concentration, resulting in increased leaf yield [61,62].

The concentration of 7.5 mM N promoted an increase in the concentration of nitric oxide, a molecule that also acts on stress signaling [11] that, together with a decrease in carbon skeletons, may have contributed to the lower synthesis of volatile substances of the specialized metabolism involved with signaling. The low percentage of defense substance, monoterpenes, could be explained by the targeting of carbon skeletons and reducing agents for the reduction and incorporation of nitrate, once that monoterpene synthesis occur in the chloroplast, sharing the same resources [28,66,67]. The high percentage of sesquiterpenes may have aided the antioxidant enzymes in controlling lipid peroxidation.

The cultivation of *Annona emarginata* revealed *trans*-caryophyllene, bicyclogermacrene, and γ-gurjunene, synthesized on the same route [68]. These substances have bactericidal activity [31,69,70] and may contribute to the defense of the species. We found a higher relative percentage of *trans*-caryophyllene when the plants are younger (150 DBT) and a higher relative percentage of bicyclogermacrene and γ-gurjunene when the plants are older (206 DBT). It appears that *A*. *emarginata* directs the resources for the synthesis of these substances according to the stage of development. These results are confirmed in the dendrogram where we observed the separation of the plants cultivated with different nitrogen levels in two clusters.

Varying the nitrogen levels in *Annona emarginata* cultivation revealed that depending on the concentration, volatile substances show higher or lower synthesis and participation in the system of signaling and defense in the plant. These results may suggest that of volatile substances participate in resistance to pests and diseases, which is a necessary condition for *Annona emarginata* to be preferentially used as rootstock for *Annona* x *atemoya*.

## Supporting information

S1 Table Treatments applied to plants of *A*. *emarginata*, submitted in Hoagland and Arnon’s nutrient solution n°1 containing macronutrients, with different nitrogen concentrations, micronutrients and iron-EDTA solution. (DOCX)

S2 Table Temperature, relative humidity, and photosynthetically active photon flow density during evaluations of *Annona emarginata* subjected to 1.87, 3.75, 5.62 and 7.5 mM N at 150, 164, 178 and 206 days after beginning treatment (DBT). (DOCX)

S1 Fig Nitrate reductase activity in *Annona emarginata* leaves grown using different nitrogen concentrations. Data are presented as the mean ± SE (n = 4). The means were compared using the Tukey’s test, with a probability level of 5%.
(DOCX)

S2 Fig Leaf amino acid concentration of *Annona emarginata* grown using different nitrogen concentrations. Data are presented as the mean ± SE (n = 4). The means were compared using Tukey’s test, with a probability level of 5%.
(DOCX)

S3 Fig (A) Total sugar (μg g FW−1), (B) reducing sugar (μg g FW−1), (C) sucrose (μg g FW−1), (D) starch (μg g FW−1) in *Annona emarginata* grown under different nitrogen concentrations. Data are presented as the mean ± SE (n = 4). The means were compared using Tukey’s test, with a probability level of 5.
(DOCX)

S4 Fig (A) Lipid peroxidation (MDA, malondialdehyde nmol g−1 of FW) (B) Superoxide dismutase (SOD, U mg−1 protein); (C) peroxidase (POX, μmol of purpurogallin min−1 mg−1 protein); and (D) catalase (CAT, μKat μg−1 protein) of *Annona emarginata* grown under different nitrogen concentrations. The means were compared using Tukey’s test, with a probability level of 5.
(DOCX)

S5 Fig (A) Net assimilation rates (NAR, dm^2^ g); (B) relative growth rates (RGR, gg^−1^day^-1^) and (C) leaf-specific weight (LSW, dm^2^ g^−1^) of *Annona emarginata* grown under different nitrogen concentrations.
(DOCX)
