# Supplementary material for: Nitrogen in the defense system of Annona emarginata (Schltdl.) H. Rainer
Source: PLoS One. 2019 Jun 6;14(6):e0217930. doi: 10.1371/journal.pone.0217930 (PMC6553785; doi:10.1371/journal.pone.0217930)
Supplement: S1 Table — (DOCX) [file pone.0217930.s001.docx]

| Treatments |  | 7.5 mM | 5.62 mM | 3.75 mM | 1.87 mM |
| --- | --- | --- | --- | --- | --- |
| Macronutrients | Stoke solution (M) | Nutrition solution (ml.L^-1^) | | | |
| KH_2_PO_4_ | 136.08 | 0.75 | 0.75 | 0.75 | 0.75 |
| KNO_3_ | 101.10 | 2.52 | 2.80 | 1.90 | 0.94 |
| Ca(NO_3_)_2_ | 236.16 | 3.75 | 2.80 | 1.90 | 0.94 |
| Mg(SO_4_).7H_2_O | 246.50 | 1.50 | 1.50 | 1.50 | 1.50 |
| (0.5M) K_2_SO_4_ | 87.13 | 0 | 0.92 | 1.82 | 2.72 |
| (0.01M) CaSO_4_ | 1.36 | 0 | 0 | 60.00 | 150.00 |
| Micronutrients | Stoke solution (g.L^-1^) | 0.75 | 0.75 | 0.75 | 0.75 |
| H_3_BO_3_ | 2.86 g |  |  |  |  |
| MnCl_2_.4H_2_O | 1.81 g |  |  |  |  |
| ZnSO_4_.7H_2_O | 0.22 g |  |  |  |  |
| CuSO_4_.5H_2_O | 0.08 g |  |  |  |  |
| H_2_MoO_4_.H_2_O | 0.02 g |  |  |  |  |
| Iron-EDTA Solution* | Stoke solution (g.L^-1^) | 0.75 | 0.75 | 0.75 | 0.75 |
| Bissodic EDTA (C_10_H_14_N_2_O_8_Na_2_.2H_2_O) | 26.10 g |  |  |  |  |
| FeSO_4_.7H_2_O | 24.90 g |  |  |  |  |

*Solution catalyzed by 265.00 mL NaOH (M)
