# Supplementary material for: Nitrogen in the defense system of Annona emarginata (Schltdl.) H. Rainer
Source: PLoS One. 2019 Jun 6;14(6):e0217930. doi: 10.1371/journal.pone.0217930 (PMC6553785; doi:10.1371/journal.pone.0217930)
Supplement: S2 Table — (DOCX) [file pone.0217930.s002.docx]

| Days after transplantation | Air temperature  (ºC) | Relative humidity (%) | Photosynthetically active photon flow density (µmol.m^-2^s^-1^) |
| --- | --- | --- | --- |
| 150 | 38.69 ± 0.40 | 30.68 ± 0.64 | 879.77 ± 47.68 |
| 164 | 35.43 ± 1.58 | 32.50 ± 1.57 | 1036.05 ± 88.04 |
| 178 | 28.19 ± 0.63 | 43.91 ± 1.27 | 773.97 ± 65.56 |
| 192 | 32.43 ± 0.30 | 29.09 ± 1.53 | 804.46 ± 41.65 |
| 206 | 32.84 ± 0.36 | 37.47 ± 0.28 | 759.78 ± 63.25 |
