# Supplementary material for: Nitrogen in the defense system of Annona emarginata (Schltdl.) H. Rainer
Source: PLoS One. 2019 Jun 6;14(6):e0217930. doi: 10.1371/journal.pone.0217930 (PMC6553785; doi:10.1371/journal.pone.0217930)
Supplement: S1 Fig — Data are presented as the mean ± SE (n = 4). The means were compared using the Tukey’s test, with a probability level of 5%. (DOCX) [file pone.0217930.s003.docx]

7.5 mM N y = 0.8315x^3^ – 73.049x^2^ + 1653.3x + 3222.9;

5.62 mM N y = 151.24x + 6690.7;

3.75 mM N y = 0.8451x^3^ – 72.733x^2^ + 1566.8x + 5370.1;

1.87 mM N y = 1013.9x + 6202.1.
