# Supplementary material for: Nitrogen in the defense system of Annona emarginata (Schltdl.) H. Rainer
Source: PLoS One. 2019 Jun 6;14(6):e0217930. doi: 10.1371/journal.pone.0217930 (PMC6553785; doi:10.1371/journal.pone.0217930)
Supplement: S2 Fig — Data are presented as the mean ± SE (n = 4). The means were compared using Tukey’s test, with a probability level of 5%. (DOCX) [file pone.0217930.s004.docx]

7.5mM N y = –0.0018x³ + 0.1485x² – 2.744x + 46.990;

5.62 mM N y = –0.01363x² + 0.728x + 48.695;

3.75 mM N y = –0.001826x³ + 0.1612x² – 3.555x + 60.065;

1.87 mM N y = 0.0007835x³ –0.0657x² – 0.03830x + 40.918.
