# Supplementary material for: Nitrogen in the defense system of Annona emarginata (Schltdl.) H. Rainer
Source: PLoS One. 2019 Jun 6;14(6):e0217930. doi: 10.1371/journal.pone.0217930 (PMC6553785; doi:10.1371/journal.pone.0217930)
Supplement: S3 Fig — (A) Total sugar (μg g FW−1), (B) reducing sugar (μg g FW−1), (C) sucrose (μg g FW−1), (D) starch (μg g FW−1) in Annona emarginata grown under different nitrogen concentrations. Data are presented as the mean ± SE (n = 4). The means were compared using Tukey’s test, with a probability level of 5. (DOCX) [file pone.0217930.s005.docx]

Equations for total sugar:

7.5mM N y = −0.001287x³ + 0.1284x² + 3.94x + 168.63;

5.62 mM N y = −0.000983x³ + 0.0786x² − 1.229x + 141.46;

3.75 mM N y = −0.002248x³ + 0.2108x² − 5.0946x + 158.08;

1.87 mM N y = −0.7695x + 172.83.

Equations for reducing sugar:

7.5mM N y = −0.00000658x³ + 0.0007608x² − 0.02364 + 0.78;

5.62 mM N y = −0.000007965x³ + 0.00078x² − 0.020x + 0.783;

3.75 mM N y = −0.0000163x³ + 0.00157x² − 0.03830x +0.79;

1.87 mM N ns.

Equations for sucrose:

7.5mM N y = 0.01428x² − 0.7587x + 92.66;

5.62 mM N ns;

3.75 mM N y = 0.01017x² − 0.4468x + 87.51;

1.87 mM N ns.

Equations for starch

7.5mM N ns;

5.62 mM N y= −0.23512x + 47.45;

3.75 mM N y = 0.13207x + 34.28;

1.87 mM N ns.
