# Supplementary material for: Nitrogen in the defense system of Annona emarginata (Schltdl.) H. Rainer
Source: PLoS One. 2019 Jun 6;14(6):e0217930. doi: 10.1371/journal.pone.0217930 (PMC6553785; doi:10.1371/journal.pone.0217930)
Supplement: S4 Fig — (A) Lipid peroxidation (MDA, malondialdehyde nmol g−1 of FW) (B) Superoxide dismutase (SOD, U mg−1 protein); (C) peroxidase (POX, μmol of purpurogallin min−1 mg−1 protein); and (D) catalase (CAT, μKat μg−1 protein) of Annona emarginata grown under different nitrogen concentrations. The means were compared using Tukey’s test, with a probability level of 5. (DOCX) [file pone.0217930.s006.docx]

Equations for lipid peroxidation:

7.5 mM N y = −1.4117x^3^ + 13.579x^2^ – 38.89x + 57.32;

5.62 mM N ns;

3.75 mM N y = 0.7707x^2^ – 4.2233x + 28.948;

1.87 mM N y = −1.6133x^3^ + 15.614x^2^ – 43.983x + 61.758.

Equations for superoxide dismutase:

7.5 mM N y = −1355.2x^3^ + 10657x^2^ − 21954x + 14705;

5.62 mM N y = −575.39x^3^ + 4619.7x^2^ − 10113x + 9674.5;

3.75 mM N y = −1533.4x^3^ + 13380x^2^ − 33407x + 27028;

1.87 mM N y = −1090x^3^ + 9315x^2^ − 22720x + 18908.

Equations for peroxidase:

7.5 mM N y = −4.1283x^3^ + 37.554x^2^ – 105.34x + 117.46;

5.62 mM N y = −3.2408x^3^ + 27.515x^2^ – 67.824x + 71.572;

3.75 mM N y = −4.586x + 42.118;

1.87 mM N y = −7.0136x^2^ + 45.762x – 25.668.

Equations for catalase:

7.5 mM N y = −2.5283x^3^ + 22.194x^2^ – 55.477x + 40.792;

5.62 mM N y = −0.7658x^3^ + 7.0039x^2^ – 18.97x + 16.374;

3.75 mM N y = −0.8075x^3^ + 7.3454x^2^ – 19.177x + 15.308;

1.87 mM N y = −3.7408x^3^ + 32.132x^2^ – 77.327x + 53.41.
