# Supplementary material for: Nitrogen in the defense system of Annona emarginata (Schltdl.) H. Rainer
Source: PLoS One. 2019 Jun 6;14(6):e0217930. doi: 10.1371/journal.pone.0217930 (PMC6553785; doi:10.1371/journal.pone.0217930)
Supplement: S5 Fig — (A) Net assimilation rates (NAR, dm2 g); (B) relative growth rates (RGR, gg−1day-1) and (C) leaf-specific weight (LSW, dm2 g−1) of Annona emarginata grown under different nitrogen concentrations. (DOCX) [file pone.0217930.s007.docx]

Equations Net assimilation rates:

7.5 mM N y = −0.0859x^2^ + 0.4164x + 0.2096;

5.62 mM N y = −0.0101x^2^ – 0.0262x + 0.7063;

3.75 mM N y = 0.0295x^2^ – 0.1695x + 0.7206;

1.87 mM N y = −0.0057x^2^ + 0.0449x + 0.3703.

Equations relative growth rates

7.5 mM N y = −1E − 08x^2^ + 0.0049x + 0.0339;

5.62 mM N y = −6E − 09x^2^ – 0.0176x + 0.084;

3.75 mM N y = −0.001x^2^ – 0.0097x + 0.0625;

1.87 mM N y = −7E − 09x^2^ – 0.0288x + 0.1001.

Equations leaf specific weight

7.5 mM N y = 0.0826x^2^ – 0.3504x + 0.8924;

5.62 mM N y = 0.0081x^2^ + 0,0045x + 0.4887;

3.75 mM N y = −0.0136x^2^ + 0.0201x + 0.691;

1.87 mM N y = 0.0712x^2^ – 0.4804x + 1.3503.
